# The epidemiology of primary headache disorders in Zambia: a population-based door-to-door survey

**DOI:** 10.1186/s10194-015-0515-7

**Published:** 2015-04-02

**Authors:** Edward Mbewe, Pachuau Zairemthiama, Hsueh-Han Yeh, Ravi Paul, Gretchen L Birbeck, Timothy J Steiner

**Affiliations:** Chainama College of Health Sciences, Lusaka, Zambia; Chikankata Hospital, Mazabuka, Zambia; Department of Epidemiology and Biostatistics, and International Neurologic and Psychiatric Epidemiology Program (INPEP), Michigan State University, East Lansing, MI USA; Department of Psychiatry, School of Medicine, University of Zambia, Lusaka, Zambia; Department of Neurology, University of Rochester, Rochester, NY USA; Department of Neuroscience, Norwegian University of Science and Technology, Edvard Griegs Gate, NO 7491 Trondheim, Norway; Division of Brain Sciences, Imperial College London, London, UK

**Keywords:** Epidemiology, Prevalence, Migraine, Tension-type headache, Medication-overuse headache, Sub-Saharan Africa, Global Campaign against Headache

## Abstract

**Background:**

Little is known of the epidemiology of primary headache disorders in sub-Saharan Africa. We performed a population-based survey in Zambia using methods previously tested in multiple other countries.

**Methods:**

This cross-sectional survey was conducted by visiting households unannounced, using cluster-randomized sampling, in the mostly urban Lusaka Province and mostly rural Southern Province. Within clusters, households were selected randomly, as was one adult member (18-65 years old) of each selected household. A structured questionnaire, translated into the local languages, was administered face-to-face by trained interviewers. Demographic enquiry was followed by diagnostic questions based on ICHD-II criteria. A random sub-sample of participants were invited for subsequent physician-interview to validate the diagnostic part of the questionnaire.

**Results:**

Of 1,134 eligible household members contacted, 1,085 (450 male, 887 urban) consented to interview (refusal rate 4.3%). Others who had been selected but remained unavailable on three visits were not counted as refusals since their reasons were unknown, but gave rise to gender biases, being mostly male in urban areas and mostly female in rural areas. Statistical correction was applied. Adjusted for gender and habitation (urban/rural), the 1-year prevalence of any headache was 61.6%, of migraine 22.9%, of tension-type headache (TTH) 22.8%, of headache on ≥15 days/month 11.5% and of probable medication-overuse headache (pMOH) 7.1%. The adjusted point-prevalence of any headache (headache yesterday) was 19.1%. There was a small proportion (5.3%) of unclassified headache, some of which may have been secondary. The overwhelmingly strong association was between urban dwelling and pMOH (OR: 8.6; P=0.0001), with an urban prevalence of 14.5% (gender-adjusted). Validation of the questionnaire was limited by participants’ reluctance to present for physician review, substantial delays in doing so and major self-selection bias among those who did. These were unavoidable problems in resource-limited Zambia.

**Conclusions:**

Primary headache disorders, common in high-income countries, are at least as prevalent in Zambia, a sub-Saharan African country. The selectively urban problem of pMOH seems likely to reflect ready availability of non-prescription analgesics, without easy access to professional health care for headache or any focused public-health education regarding correct usage of analgesics or the dangers of their overuse.

## Background

Worldwide, headache disorders are among the most common medical conditions. According to the Global Burden of Disease Study 2010 (GBD 2010), tension-type headache (TTH) and migraine are respectively the second and third most prevalent diseases globally (behind dental caries) [1,2]. Migraine is the seventh leading specific cause of years of life lost to disability (YLDs), responsible for 2.9% of all YLDs, and more than half of all YLDs attributable to neurological disorders [1-3]. Both migraine and TTH can lead, through mistreatment, to medication-overuse headache (MOH), which by definition occurs on ≥15 days/month and is a major contributor to disability burden at the individual level [4].

GBD 2010 relied upon epidemiological evidence from all regions of the world. There were, however, considerable gaps in this knowledge; this was true for all world regions, but particularly so in several [5]. In sub-Saharan Africa (SSA), as a case in point, there had been few studies of prevalence of primary headache disorders, most of these had been in select sub-populations and none had assessed burden [6-14]. Prevalence estimates, perhaps not surprisingly, had varied considerably: in Zimbabwe, Quesada-Vazquez *et al.* reported a primary headache disorder in 37.1% of psychiatric hospital workers [7]; in rural Ethiopia, Takele *et al.* recorded headache in 16.4% of mill workers [8]; in a rural community of Benin, Houinato *et al.* estimated a 3.3% lifetime prevalence of migraine [11]; and in northern Tanzania Winkler *et al.* reported a 7.0% 1-year prevalence of TTH and 4.3% of migraine [10,12]. This range of variation undoubtedly reflected differences in methodology and, especially, case definition, as well as in the selected populations surveyed [15]. These estimates were, nonetheless, consistent in one respect: all were considerably lower than global averages [5].

The aim of this study, a project within the Global Campaign against Headache [16-19], was to fill the knowledge gap regarding primary headache disorders in the general population of Zambia. It was the first such study in the African Region, and undertaken primarily to inform health policy-makers. Understanding the scale and scope of the burden of headache disorders is the basis of health-needs assessment, of planning effective health services and of appropriate resource-allocation for what is and should be seen as a public-health priority [20]. We estimated 1-year prevalence of headache, migraine, TTH, all causes of headache on ≥15 days/month, MOH (as probable MOH [pMOH]) and point (1-day) prevalence of headache (“headache yesterday”); these are reported here. We also assessed disability and other aspects of burden attributable to these disorders, which will be described elsewhere.

## Methods

### Ethics

This study was approved by the University of Zambia’s Research Ethics Committee. Verbal informed consent was obtained from key informants and all participants before we asked the survey questions.

### Study design

This was a cross-sectional questionnaire survey of adults aged 18–65 years, conducted door-to-door. Participants were randomly selected from two of the nine provinces of Zambia: the mostly urban population in Lusaka Province, and the mostly rural population in the Southern Province.

### Instrument

We used a local culturally-adapted version of the structured questionnaire [21] developed for these surveys by *Lifting The Burden* (LTB) [16-19] and employed previously in Russia [22], China [23] and India [24] and subsequently in a total of 19 countries in 18 languages [21]. The questionnaire was translated according to LTB’s translation protocol for lay documents [25] from English into three local languages: Bemba and Nyanja for Lusaka Province, and Tonga for the Southern Province.

The questionnaire was composed of five parts: personal and demographic enquiry, and headache screening questions, which were addressed to all respondents; these were followed in those screening positively by diagnostic questions, enquiry into burden and questions on selected comorbidities.

The screening question for headache was: “*In the last year, have you had headache that was not part of another illness?*” Participants who answered “no” were classified as headache-free; those who answered “yes” were asked if all their headaches were of one or more types and, if more than one, to focus in the subsequent questions on the one that was most bothersome. Only that headache was diagnosed. The point prevalence of headache was estimated by asking: “*Did you have a headache yesterday?*”

### Selection and training of interviewers

In Lusaka Province, interviewers were interested faculty and advanced students from Chainama College of Health Sciences. In the Southern Province, interviews were conducted by the Chikankata Epilepsy Care team, whose staff had been conducting community- and hospital-based research for over a decade.

All interviewers attended a 3-day training session at Chainama Hills College Hospital, Lusaka. Training included clinical aspects of headache disorders and the theoretical and practical aspects of the study design and purpose and application of the questionnaire. The interviewers were then assessed in supervised interviews.

### Pre-pilot and pilot surveys

A clinic-based, pre-pilot study was conducted in two urban health centres in Lusaka. The original English-language version of the draft questionnaire was administered by physicians, clinical officers or nurses, translated at point of application, to 20 adults aged 18–65 years in an approximately equal mix of patients presenting with headache and others with unrelated disorders. The purpose was to establish that questions were acceptable and inoffensive. This exercise guided local cultural adaptation of the questionnaires, and led to a final draft.

The pilot survey was community-based, conducted in both rural and urban areas using the translated final-draft questionnaires over the course of two months. Convenient communities were identified in the two provinces, and 10 adults aged 18–65 years were selected from each by a mixture of convenience and purposive sampling. Thus a total of 20 adults were interviewed by physicians, clinical officers or nurses. The purpose was to test the translated questionnaires, in the field, for feasibility. Final adaptations were made based upon feedback from this exercise.

### Sampling, and main survey

The main study used cluster-sampling followed by simple random sampling of dwellings and of one adult participant within each household’s family.

Sampling was facilitated by previously-collected census data providing neighbourhood blocks and household locations. In each urban or rural area, the interviewers randomly selected blocks or circumscribed collections of dwellings (clusters), and then one or more dwellings within each of these. When the door to a selected dwelling was not answered at first visit, two further visits were made on subsequent days before the dwelling was excluded and replaced by another in the block.

Within selected dwellings, each non-biologically related family was a sampling unit. A key informant was asked to list all members aged 18–65 years within the family. This informant was preferably the female head-of-household, who traditionally takes responsibility for and is most knowledgeable of other household members’ health. The Kish grid method [26] was applied to select one member from this list for interview. When that person was present, he or she was asked the demographic and screening questions; when not, the female head-of-household would answer them on that person’s behalf. When the screening question was answered positively (*ie*, headache was reported in the last year), and the person was present, the full interview was conducted immediately; otherwise, an appointment was made for a return visit when the person was expected to be at home. Any selected respondent who remained unavailable after three visits was replaced from another household.

Data collection in the field was quality-assured by EM, who made random unannounced checks of interviewers’ work in the field.

### Validation

A sub-sample of 50 participants from each province were randomly selected for validation of the diagnostic questionnaire. With only two full-time adult neurologists to serve all the clinical, administrative and educational needs of this country of >13 million people, specialist-level evaluation for the validation study was not possible. Two physicians, a general medical officer in the rural region (PZ) and a psychiatrist in the urban (RP), received additional training from a neurologist (GB) in headache diagnosis according to ICHD-II [27]. They were blinded to the participants’ questionnaire responses, and used their clinical judgement as well as ICHD-II criteria to make diagnoses deemed to be the gold standard. The goal was to complete these evaluations within 1 month of the participants’ completion of the questionnaire.

### Data management

Completed paper questionnaires and physicians’ evaluations were transported to the central research office for data entry into Microsoft Excel and eventual importation into SAS. A 20% random check of records was undertaken to assure data-entry quality with an error rate of <2% identified. Paper records were stored securely for quality checks and data confirmation.

### Analysis and statistics

Age in years was analyzed as a categorical variable (18–29, 30–39, 40–49, 50–65). Marital status was classified as single, married, widowed or divorced. Educational level was classified as primary (grades 1–7), secondary (grades 8–12) or higher education (college or university). Employment status was categorized as unemployed, or as unskilled, skilled or professional work. Income per capita per month was categorized as below or above the poverty line (~ USD 40; at the time of the survey, USD 1 = ZMK 5,173) [28].

Interviewers did not make diagnoses. These were derived during analysis, algorithmically [21], from the questionnaire responses. Participants reporting headache on ≥15 days/month were first separated and described as a separate group because they cannot be adequately diagnosed by questionnaire [21]. However, those with headache on ≥15 days/month who also reported regular use of headache medication on >3 days/week were considered to have pMOH. To all others, the algorithm applied ICHD-II criteria in the order: migraine, TTH, probable migraine, probable TTH [27]. Cases of migraine and probable migraine, and of TTH and probable TTH, were then combined for prevalence estimation and further analyses [15]. Remaining cases were unclassified.

We recorded headache frequency in days over the preceding 3 months, and typical headache intensity on a verbal rating scale (“not bad”, “quite bad” and “very bad”). The latter ratings were transformed into a numerical scale 1–3, which was treated as a continuous variable.

For the validation exercise comparing questionnaire-derived and physicians’ diagnoses, sensitivity, specificity and positive (PPV) and negative predictive values (NPV) were calculated with 95% confidence intervals (CIs). We used Cohen’s kappa coefficient to estimate overall agreement between diagnoses.

Analyses were performed with SAS version 9.2 (SAS Institute Inc, Cary, NC, USA) or Excel 2007 (Microsoft Corporation, Redmond, WA, USA). We calculated *P*-values as an aid to interpretation. Chi-squared, Fisher’s exact and binomial proportion tests were applied to compare distributions and proportions. We used logistic regression analysis to examine associations between demographic variables and prevalence, calculating odds ratios (ORs) and adjusted ORs with 95% CIs.

## Results

Of 1,134 household members contacted in the main survey, 1,085 (450 male, 635 female; 198 rural [Southern Province], 887 urban [Lusaka Province]) consented to be interviewed (refusal rate 4.3%). They were selected from 3,035 enumerated eligible adults (1,448 male, 1,587 female) in these households. The sociodemographic characteristics of participants are displayed in Table 1. The male:female ratio of our sample (41.5:58.5) diverged from the national ratio (very close to 50:50) [29]. The sample also did not match the urban/rural distribution (40:60) of the Zambian population [29]. Adjustments to observed prevalences were therefore necessary for both gender and habitation, and are reported below. Rural participants were more often male, younger, less well educated, more likely to be unemployed, less likely to be in skilled or professional work and on lower incomes than urban participants. However, marital status, education level, employment status and income levels in our samples were consistent with those of the Lusaka and Southern Province populations [29].

**Table 1:** **Demographic characteristics of the study sample**

	**All n (%)**	**Rural n (%)**	**Urban n (%)**	***P*** **-value** ^**a**^
**Total**	1,085 (100.0)	198 (100.0)	887 (100.0)	
**Gender**				<0.0001
Female	635 (58.5)	75 (37.9)	560 (63.1)	
Male	450 (41.5)	123 (62.1)	327 (36.9)	
**Age** (years)				0.0004
18-29	370 (34.1)	93 (47.0)	277 (31.2)	
30-39	361 (33.3)	51 (25.8)	310 (35.0)	
40-49	207 (19.1)	33 (16.7)	174 (19.6)	
50-65	147 (13.5)	21 (10.6)	126 (14.2)	
**Marital status**				<0.0001
Single	310 (28.6)	72 (36.4)	238 (26.8)	
Married	549 (50.6)	108 (54.6)	441 (49.7)	
Widowed	114 (10.5)	13 (6.6)	101 (11.4)	
Divorced	110 (10.1)	5 (2.5)	105 (11.8)	
**Educational level**				<0.0001
None/primary	270 (24.9)	71 (35.9)	199 (22.4)	
Secondary	584 (53.8)	78 (39.4)	506 (57.1)	
Higher	225 (20.7)	49 (24.8)	176 (19.8)	
**Employment**				<0.0001
Unemployed	476 (43.9)	117 (59.1)	359 (40.5)	
Unskilled	282 (26.0)	51 (25.8)	231 (26.0)	
Skilled	174 (16.0)	19 (9.6)	155 (17.5)	
Professional	137 (12.6)	11 (5.6)	126 (14.2)	
**Income per month**				<0.0001
≤ USD 40	258 (23.8)	121 (61.1)	137 (15.5)	
> USD 40	827 (76.2)	77 (38.9)	750 (84.6)	

^a^ *P*-values (Chi-squared) compared distributions within the variable between rural and urban participants.

Prevalence overall and by age, gender and habitation is set out in Table 2. In total, 781 participants (72.0%; males 66.2%, females 76.1%) reported headache unrelated to another illness in the past year, and 307 (28.3%; males 21.3%, females 33.1%) reported headache on the day prior to the interview (headache yesterday). Any headache in the last year (75.7% *vs* 52.1%, gender-adjusted) and headache yesterday (30.4% *vs* 11.5%, gender-adjusted) were both more prevalent among urban than rural dwellers. Adjusted for gender and habitation, the 1-year prevalence of any headache was 61.6% and the point prevalence (headache yesterday) was 19.1%. Point prevalence increased consistently with age (Table 2), an association which appeared to be driven by pMOH (see below).

**Table 2:** **Observed 1-year prevalence (% [95% confidence interval]) by gender, age and habitation, and by headache type**

	**Migraine (n = 253)**	**TTH (n = 263)**	**Any headache on ≥15d/m (n = 207)**	**pMOH (n = 138)**	**Any headache yesterday (n = 307)**
**All**	23.3 [20.9-25.9]	24.2 [21.8-26.9]	19.1 [16.9-21.5]	12.7 [10.9-14.8]	28.3 [25.7-31.1]
**Gender**					
Male	18.0 [14.7-21.8]	27.8 [23.8-32.1]	14.9 [11.9-18.5]	9.3 [7.0-12.4]	21.3 [17.8-25.3]
Female	27.1 [23.8-30.7]	21.7 [18.7-25.1]	22.1 [19.0-25.4]	15.1 [12.5-18.1]	33.1 [29.5-36.8]
**Age (yr)**					
18-29	24.9 [20.7-29.5]	24.3 [20.2-29.0]	14.6 [11.3-18.6]	7.0 [4.8-10.1]	24.9 [20.7-29.5]
30-39	22.1 [18.1-26.7]	27.7 [23.3-32.5]	17.5 [13.9-21.7]	12.5 [9.4-16.3]	28.0 [23.6-32.8]
40-49	26.6 [21.0-33.0]	23.2 [17.9-29.4]	20.2 [15.4-26.3]	15.5 [11.1--21.1]	30.0 [24.1-36.5]
50-65	17.7 [12.3-24.7]	17.0 [11.7-26.5]	32.7 [25.6-40.6]	23.8 [17.6-31.3]	34.7 [27.5-42.7]
**Habitation**					
Rural	21.7 [16.5-28.0]	22.7 [17.4-29.1]	4.0 [1.9-7.9]	2.0 [0.6-5.3]	11.6 [7.8-16.9]
Urban	23.7 [21.0-26.6]	24.6 [21.9-27.5]	22.4 [19.8-25.3]	15.1 [12.9-17.6]	32.1 [29.1-35.3]

TTH: tension-type headache; pMOH: probable medication-overuse headache; d/m: days/month.

The observed 1-year prevalence of migraine was 23.3% (12.7% definite, 10.6% probable), with a female preponderance of about 3:2 (Table 2) (gender- and habitation-adjusted: 22.9%). The observed 1-year prevalence of TTH was 24.2% (16.8% definite, 7.4% probable), with a male preponderance of about 4:3 (gender- and habitation-adjusted: 22.8%). Migraine prevalence peaked during the ages 40–49 years, then dropped to its lowest level in those aged 50–65 years. TTH was most common in those aged 30–39 and least common in those over 50. There was a small proportion (5.3%) of unclassified episodic headache, seen more or less equally in both genders and all age groups.

Headache on ≥15 days/month was reported by 207 participants (19.1%; male:female about 2:3) and diagnosed as pMOH in 12.7% (male:female about 3:5) (Table 2). Prevalence of pMOH increased steadily with age; prevalence of other headache on ≥15 days/month fluctuated, but again was highest in those aged 50–65 years (Table 2). A far larger proportion of urban (22.4%) than of rural participants (4.0%; *P* < 0.05 [binomial proportional test]) had headache on ≥15 days/month, and a very marked difference was observed in the prevalence of pMOH (urban 15.1% [gender-adjusted: 14.5%]; rural 2.0% [gender-adjusted: 2.1%]; *P* < 0.05). Bivariate analysis confirmed the very strong association between pMOH and urban dwelling (OR = 8.6 [95% CI 3.2-23.6]; *P* = 0.0001) (Table 3). The gender- and habitation-adjusted prevalence of pMOH was 7.1% and of other headache on ≥15 days/month was 4.4%.

**Table 3:** **Bivariate analysis of associations with each diagnosis adjusted for gender and age**

	**Migraine**		**Tension-type headache**		**pMOH**	
	**OR**	***P***	**OR**	***P***	**OR**	***P***
**Habitation**						
Rural	reference		reference		reference	
Urban	1.1 [0.77-1.6]		1.1 [0.77-1.6]		8.6 [3.2-23.6]	0.0001
**Education**						
None/primary	reference		reference		reference	
Secondary	1.4 [0.95-2.0]		0.46 [0.34-0.64]	<0.0001	0.59 [0.38-0.90]	0.0147
College or university	2.1 [1.4-3.2]	0.0004	0.22 [0.14-0.37]	<0.0001	1.1 [0.71-1.8]	
**Employment**						
None	reference		reference		reference	
Unskilled	1.2 [0.84-1.7]		1.5 [1.1-2.1]	0.0149	1.6 [1.1-2.6]	0.0232
Skilled	1.1 [0.83-1.6]		1.6 [1.2-2.2]	0.0010	1.5 [1.03-2.3]	0.0343
Professional	2.1 [1.4-3.2]	0.0005	0.46 [0.26-0.82]	0.0080	1.5 [0.91-2.7]	
**Income per month (USD)**						
≤40	reference		reference		reference	
>40	1.3 [0.88-1.8]		1.3 [0.93-1.9]		6.0 [2.8-13.1]	<0.0001

pMOH: probable medication-overuse headache; OR: odds ratio with 95% confidence interval in parenthesis.

We also used bivariate analysis to examine associations with socioeconomic indicators (Table 3). Increasing educational level was quite strongly associated with more migraine and less TTH (ORs respectively of 2.1 and 0.22 for those attending college or university) but had no clear relationship with pMOH. This was well reflected in employment, with professionals showing ORs for migraine and TTH of 2.1 and 0.46 compared with those not employed. All employed groups were somewhat more likely (OR ~ 1.5) to have pMOH. In bivariate analysis, higher income (above USD 40 per month) was weakly and non-significantly associated with migraine and TTH but strongly and significantly with pMOH (OR = 6.0; *P* < 0.0001). Higher income was also strongly associated with urban dwelling (*P* < 0.0001) (Table 1), and the association between higher income and pMOH did not survive multivariate analysis in the logistic regression model.

Headache frequency overall was high (mean 10.3 days/month), creating among those with headache a probability of headache on any particular day of 0.34. Headache frequency averaged 3.4 days/month among people with migraine and 2.5 days/month among those with TTH, while participants diagnosed with pMOH had headache virtually every day. Three quarters (74.2%) of participants with migraine and over two thirds (69.6%) with pMOH, but only 13.3% with TTH, reported “very bad” headaches. Since headache intensity ratings were transformed into a numerical continuous variable from 1 (“not bad”) to 3 (“very bad”), the means were 2.7 for migraine, 2.6 for pMOH, and 1.9 (implying moderate intensity) for TTH.

### Validation

Household occupants were generally willing to participate in the main survey, conducted at home. However, physician-evaluation for the validation exercise required travel to a local health-care facility, and there was significant reluctance among invited participants, especially within the urban population, to make this effort. More than half declined outright; others agreed but did not appear for their appointments. Among those who arrived, >90% presented >1 month after their initial interview, some as late as 4 months. Many came with complaints of recent worsening in headache frequency and/or severity, and were primarily motivated to seek health-care services. An inevitable consequence was that the validation sub-sample were, through self-selection, considerably at odds diagnostically (by questionnaire) with the overall sample (Table 4).

**Table 4:** **Comparison of questionnaire diagnoses in survey sample and validation sub-sample**

	**Survey sample (n = 1,085)**	**Validation sub-sample (n = 99)**	***P*** **-value** ^**a**^
No headache (%)	304 (28.0)	0 (0.0)	<0.0001
Unclassified headache (%)	58 (5.3)	10 (10.1)	0.0670
Migraine (%)	253 (23.3)	45 (45.5)	<0.0001
TTH (%)	263 (24.2)	22 (22.2)	0.7137
pMOH (%)	138 (12.7)	22 (22.2)	0.0131

TTH: tension-type headache; pMOH: probable medication-overuse headache.

^a^ *P*-values (Fisher’s exact test) compared the difference in proportions with the diagnosis between survey and validation samples.

Within the participants from the validation sub-sample who were actually reassessed, the questionnaire appeared more sensitive in diagnosing migraine (sensitivity: 48.0%) and more specific for TTH (specificity: 81.0%). However, the kappa coefficients demonstrated poor agreement between physicians’ and questionnaire diagnoses (Table 5).

**Table 5:** **Diagnostic validation exercise (findings derived from comparisons between questionnaire-diagnoses and physician-diagnoses made up to four months later in self-selecting participants from the validation sub-sample) (see text)**

	**Migraine**	**TTH**
Sensitivity*	48.0 [34.2-61.9]	27.8 [13.2-42.4]
Specificity*	57.1 [43.3-71.0]	81.0 [71.3-90.7]
PPV*	53.3 [38.8-67.9]	45.5 [24.7-66.3]
NPV*	51.9 [38.5-65.2]	66.2 [55.7-76.8]
Kappa	5.14%	9.52%

*Values are shown with 95% confidence intervals in parentheses.

## Discussion

This study was the first population-based survey to estimate the prevalence of primary headache disorders in Zambia, and one of the first in SSA. It employed established methods [15] and questionnaire [21-24]; it included diverse regions, employed an appropriate mix of cluster-sampling and simple random sampling, and applied ICHD-II diagnostic criteria [27]. These were considerable strengths.

Yet studies of this nature are challenging in a country like Zambia, with insufficiencies in health-care infrastructure and personnel. We intended to recruit a larger sample, based on expectations of lower prevalences of all headache types [6-14], but in fact achieved an adequate overall sample size (>1,000) in view of the prevalences actually observed. We did not, however, balance the sample well between urban and rural areas. Surveying the latter was far more demanding of resources (human more than financial), and our interviewers found it difficult against competing demands to spend the necessary time travelling. This imbalance was a significant study limitation, even though statistical correction could be applied.

We also did not achieve a good gender balance despite random sampling at household level and a very low refusal rate (4.3%) among household members actually contacted. There was a slight overall female preponderance of 52.3% among the 3,035 enumerated eligible adults, but an additional explanation must be found. Potential respondents selected for interview who remained unavailable on three visits were replaced from other households; they were not counted as refusals since their reasons for being unavailable were unknown. Unfortunately the interviewers failed to keep records of these people, but those away from the home were mostly male in urban areas and mostly female in rural areas, and it is likely that this was how the gender biases arose. Again, statistical correction could be applied, but the concern here is that failure of certain respondents to be available might reflect (lack of) interest-bias. In fact, the opposite could be the case: the screening question was commonly answered by the female head-of-household and, when negative, absence of the participant was not an issue. In other words, a selected respondent *without* headache had a somewhat higher probability of being included in the survey than one with headache.

Among the countries where the methodology had previously been employed were India [30] and Pakistan [31], which have many similar problems. Unlike these two countries, Zambia has an almost total lack of neurologists, which made application of a “gold standard” very difficult for the purpose of the diagnostic validation exercise. Additionally, physicians are too few in Zambia to justify, ethically or logistically, sending them into the communities to conduct validation assessments. As a consequence, people in the validation sub-sample who undertook the travel to a local health-care facility were highly self-selecting (45.5% migraine, 22.2% pMOH), and this factor undoubtedly explained the high proportion with more troublesome headache, likely also to be diagnostically difficult. More problematic than this, there were unwanted delays of months between survey and physician evaluations, allowing the possibility of real change in the headache disorder. Community health workers were asked why participants were so reluctant to take advantage of the opportunity for free physician evaluation. Inconvenience and travel distances were cited, but the key issue was the stigma attached to seeking services at health-care facilities associated with psychiatric illness. The structure of health-care services in Zambia mirrors the World Health Organization organizational model: health-care personnel and institutions responsible for neurological care (and therefore for performing the validation examinations) were also those providing mental-health services. The stigma of mental illness in Africa has been well described [32,33]. Ultimately, the validation exercise was unreliable, rather than the diagnostic questionnaire. It was disappointing that we could not prove its validity in the three local languages, but the questionnaire already had a record of successful use in many countries and cultures [21-24].

The reported 1-year prevalence of all headache was 72.0% (gender- and habitation-adjusted 61.6%), of migraine 23.3% (22.9%), of TTH 24.2% (22.8%), of headache on ≥15 days/month 19.1% (11.5%) and of pMOH 12.7% (7.1%). Globally, 47% of adults have been estimated to experience headache at least once within a year [5], with the most recent prevalence estimates coming from GBD 2010 for migraine (14.7%) and TTH (20.1%) [2]. No reliable global estimate is yet available for pMOH, because so few studies have been conducted and case-ascertainment is difficult [34], but a recent review found that estimates clustered around 1–1.5% [35] while all headache on ≥15 days/month may affect 3% of adults [36]. Comparisons with epidemiological studies elsewhere, using the same methods and questionnaire, put the prevalence estimate for migraine in Zambia towards the upper end of the range of these studies (India 25.2% [unpublished], Russia 20.8% [37], China 9.3% [38]) and within the range for TTH (India 35.1% [unpublished], Russia 30.8% [37], China 10.8% [38]). Therefore our Zambian data are in contradiction of previous studies in SSA (surveying less representative populations) which reported substantially lower estimates for both migraine (3.3% in rural Benin [11]) and TTH (7% in northern Tanzania [10]). Primary headache is at least as common in Zambia as in the rest of the world, which carries a very important public-health message for this country and probably the entire region.

All types of headache were more common in urban areas. For migraine and TTH the association between headache and urbanicity was weak and insignificant, but for headache on ≥15 days/month it was very strong (Table 2). We noted earlier that rural participants were less well educated and on lower incomes than urban participants, which might be expected to *increase* the prevalence of headache [39-42] and therefore show the opposite effect. On the other hand, people in rural Zambia are probably more physically active, with less exposure to processed food and lower rates of obesity – trends that are reversed in more developed countries, where the poor are disproportionately exposed to physical inactivity, high-calorie low-nutrient diets, obesity and diabetes [43-45]. This may be telling us something about risk factors for headache, which perhaps will increase as the world becomes ever-more urbanized.

The striking finding in this study, of course, was the high prevalence of pMOH (gender- and habitation-adjusted: 7.1%), which compares with the global range of up to 7% but with most estimates within 1–1.5% [5,35]. While explanation is called for, clinical studies rather than epidemiological are needed to provide it. Meanwhile we can suggest the following as likely: the limited access to health care, and the limited expertise in management of headache disorders among the few health-care workers who are available, lead to a culture of recourse to analgesics obtained over-the-counter, which is unrestrained by any public health-education. Escalating use follows, this being the behaviour typically leading to MOH everywhere. There is convincing support for this from the urban/rural difference: while the prevalence of pMOH in rural areas (2.1% gender-adjusted) is high but not especially so in global terms [35], it is totally eclipsed by the egregious, and alarming, urban prevalence of 14.5% (gender-adjusted). We would expect an urban/rural difference: the very limited access to over-the-counter medication prevents such recourse to it in rural areas.

The high prevalence of pMOH largely drove the notably high mean headache frequency overall (10.3 days/month, whereas both migraine and TTH occurred, on average, on <1 day/week). This created a probability of headache on any particular day among those with headache of 0.34, and a predicted 1-day prevalence of 24.5% (0.34*72%). The reported prevalence of headache yesterday was a very compatible 28.3%, which shows two things: it affirms the veracity of these findings, especially with regard to the high-frequency headache, and it demonstrates the worth of epidemiological enquiry into headache yesterday.

The proportion of unclassified headache was not unduly high (5.3%), but we will say something about it. It was quite constant across both genders and all ages. Diagnoses were made algorithmically, applying, in order, ICHD-II criteria for migraine, TTH, probable migraine and probable TTH [15,27], having first separated participants with headache on ≥15 days/month. These 5.3% of participants therefore described headache on <15 days/month meeting none of these criteria. The questionnaire was not designed to capture secondary headache disorders, and, although the screening question (“*In the last year, have you had headache that was not part of another illness?*”) endeavoured to exclude these, it might not have succeeded if the underlying illness had not been diagnosed, or causation recognised. In Zambia, an obvious possibility was headache attributed to malaria. We should add that the last part of this screening question is not now recommended, because respondents might wrongly attribute headache to another illness and be inappropriately excluded without further enquiry [15]. The high prevalence of reported headache suggests this did not happen often, if at all.

## Conclusions

Primary headache disorders are no less common in Zambia than in the rest of the world, where they are in the top 10 causes of disability. Health policy-makers need to be aware of this. There is a major problem of headache on ≥15 days/month, largely consisting of pMOH; the latter, in theory, is entirely avoidable, and the urban/rural divide supports this. Public education regarding the risk of MOH is needed, and could be provided at relatively low cost [46]. Other countries in SSA are likely to have similar burdens of headache-related ill health.

## Abbreviations

CI: Confidence interval
GBD: Global burden of disease
ICHD: International classification of headache disorders
LTB: *Lifting The Burden*
MOH: Medication-overuse headache
OR: Odds ratio
pMOH: Probable MOH
SSA: Sub-Saharan Africa
TTH: Tension-type headache
YLD: Year of life lost to disability

## References

[1] Murray CJ, Vos T, Lozano R, Naghavi M, Flaxman AD, Michaud C, Ezzati M, Shibuya K, Salomon JA, Abdalla S, Aboyans V, Abraham J, Ackerman I, Aggarwal R, Ahn SY, Ali MK, Alvarado M, Anderson HR, Anderson LM, Andrews KG, Atkinson C, Baddour LM, Bahalim AN, Barker-Collo S, Barrero LH, Bartels DH, Basáñez MG, Baxter A, Bell ML, Benjamin EJ (2013). Disability-adjusted life years (DALYs) for 291 diseases and injuries in 21 regions, 1990–2010: a systematic analysis for the Global Burden of Disease Study 2010. Lancet.

[2] Vos T, Flaxman AD, Naghavi M, Lozano R, Michaud C, Ezzati M, Shibuya K, Salomon JA, Abdalla S, Aboyans V, Abraham J, Ackerman I, Aggarwal R, Ahn SY, Ali MK, Alvarado M, Anderson HR, Anderson LM, Andrews KG, Atkinson C, Baddour LM, Bahalim AN, Barker-Collo S, Barrero LH, Bartels DH, Basáñez MG, Baxter A, Bell ML, Benjamin EJ, Bennett D (2012). Years lived with disability (YLDs) for 1160 sequelae of 289 diseases and injuries 1990–2010: a systematic analysis for the Global Burden of Disease Study 2010. Lancet.

[3] Steiner TJ, Stovner LJ, Birbeck GL (2013). Migraine: the seventh disabler. J Headache Pain.

[4] Linde M, Gustavsson A, Stovner LJ, Steiner TJ, Barré J, Katsarava Z, Lainez JM, Lampl C, Lantéri-Minet M, Rastenyte D, Ruiz de la Torre E, Tassorelli C, Andrée C (2012). The cost of headache disorders in Europe: the Eurolight project. Eur J Neurol.

[5] Stovner LJ, Hagen K, Jensen R, Katsarava Z, Lipton RB, Scher AI, Steiner TJ, Zwart J-A (2007). The global burden of headache: a documentation of headache prevalence and disability worldwide. Cephalalgia.

[6] Dent W, Spiss H, Helbok R, Matuja W, Scheunemann S, Schmutzhard E (2004). Prevalence of migraine in a rural area in South Tanzania: a door-to-door survey. Cephalalgia.

[7] Quesada-Vazquez AJ, Rodriguez-Santana N (2006). The prevalence of primary headaches in the working population at a psychiatric hospital in Zimbabwe. Rev Neurol.

[8] Takele GM, Tekle Haimanot R, Martelletti P (2008). Prevalence and burden of primary headache in Akaki textile mill workers, Ethiopia. J Headache Pain.

[9] Ojini FI, Okubadejo NU, Danesi MA (2009). Prevalence and clinical characteristics of headache in medical students of the University of Lagos, Nigeria. Cephalalgia.

[10] Winkler A, Stelzhammer B, Kerschbaumsteiner K, Meindl M, Dent W, Kaaya J, Matuja W, Schmutzhard E (2009). The prevalence of headache with emphasis on tension-type headache in rural Tanzania: a community-based study. Cephalalgia.

[11] Houinato D, Adoukonou T, Ntsiba F, Adjien C, Avode DG, Preux PM (2010). Prevalence of migraine in a rural community in south Benin. Cephalalgia.

[12] Winkler A, Dent W, Stelzhammer B, Kerschbaumsteiner K, Meindl M, Kaaya J, Matuja W, Schmutzhard E (2010). Prevalence of migraine headache in a rural area of northern Tanzania: a community-based door-to-door survey. Cephalalgia.

[13] Dent W, Stelzhammer B, Meindl M, Matuja WBP, Schmutzhard E, Winkler AS (2011). Migraine attack frequency, duration, and pain intensity: disease burden derived from a community-based survey in northern Tanzania. Headache.

[14] Gelaye B, Peterlin BL, Lemma S, Tesfaye M, Berhane Y, Williams MA (2013). Migraine and psychiatric comorbidities among sub-Saharan African adults. Headache.

[15] Stovner LJ, Al Jumah M, Birbeck GL, Gururaj G, Jensen R, Katsarava Z, Queiroz LP, Scher AI, Tekle-Haimanot R, Wang SJ, Steiner TJ (2014). The methodology of population surveys of headache prevalence, burden and cost: principles and recommendations from the Global Campaign against Headache. J Headache Pain.

[16] Steiner TJ (2004). Lifting the burden: the global campaign against headache. Lancet Neurol.

[17] Steiner TJ (2005). *Lifting The Burden*: the global campaign to reduce the burden of headache worldwide. J Headache Pain.

[18] Steiner TJ, Birbeck GL, Jensen R, Katsarava Z, Martelletti P, Stovner LJ (2010). *Lifting The Burden*: the first 7 years. J Headache Pain.

[19] Steiner TJ, Birbeck GL, Jensen R, Katsarava Z, Martelletti P, Stovner LJ (2011). The Global Campaign, World Health Organization and *Lifting The Burden*: collaboration in action. J Headache Pain.

[20] World Health Organization, Lifting The Burden (2011). Atlas of headache disorders and resources in the world 2011.

[21] Steiner TJ, Gururaj G, Andrée C, Katsarava Z, Ayzenberg I, Yu S-Y, Al Jumah M, Tekle-Haimanot R, Birbeck GL, Herekar A, Linde M, Mbewe E, Manandhar K, Risal A, Jensen R, Queiroz LP, Scher AI, Wang SJ, Stovner LJ (2014). Diagnosis, prevalence estimation and burden measurement in population surveys of headache: presenting the HARDSHIP questionnaire. J Headache Pain.

[22] Ayzenberg I, Katsarava Z, Mathalikov R, Chernysh M, Osipova V, Tabeeva G, Steiner TJ (2011). The burden of headache in Russia: validation of the diagnostic questionnaire in a population-based sample. Eur J Neurol.

[23] Yu SY, Cao XT, Zhao G, Yang XS, Qiao XY, Fang Y-N, Feng J-C, Liu R-Z, Steiner TJ (2011). The burden of headache in China: validation of diagnostic questionnaire for a population-based survey. J Headache Pain.

[24] Rao GN, Kulkarni GB, Gururaj G, Rajesh K, Subbakrishna DK, Steiner TJ, Stovner LJ (2012). The burden of headache disorders in India: methodology and questionnaire validation for a community-based survey in Karnataka State. J Headache Pain.

[25] Peters M, Bertolote JM, Houchin C, Kandoura T, Steiner TJ (2007). Translation protocol for lay documents. J Headache Pain.

[26] Kish L (1949). A procedure for objective respondent selection within the household. JASA.

[27] Headache Classification Subcommittee of the International Headache Society (2004). The International Classification of Headache Disorders: 2nd edition. Cephalalgia.

[28] Ravallion M, Chen S, Sangraula P (2009). Dollar a day revisited. World Bank Economic Review.

[29] Central Statistical Office, Zambia. Available at http://www.zamstats.gov.zm/ (accessed on 1 September 2014).

[30] Gururaj G, Kulkarni GB, Rao GN, Subbakrishna DK, Stovner LJ, Steiner TJ (2014). Prevalence and sociodemographic correlates of primary headache disorders: results of a population-based survey from Bangalore, India. Indian J Publ Health.

[31] Herekar AD, Herekar AA, Ahmad A, Uqaili UL, Ahmed B, Effendi J, Alvi SZ, Steiner TJ (2013). The burden of headache disorders in Pakistan: methodology of a population-based nationwide study, and questionnaire validation. J Headache Pain.

[32] Sartorius N (2007). Stigma and mental health. Lancet.

[33] Saxena S, Thornicroft G, Knapp M, Whiteford H (2007). Resources for mental health: scarcity, inequity, and inefficiency. Lancet.

[34] Steiner TJ (2014). Can we know the prevalence of MOH?. Cephalalgia.

[35] Westergaard ML, Hansen EH, Glumer C, Olesen J, Jensen RH (2014). Definitions of medication-overuse headache in population-based studies and their implications on prevalence estimates: A systematic review. Cephalalgia.

[36] Jensen R, Stovner LJ (2008). Epidemiology and comorbidity of headache. Lancet Neurol.

[37] Ayzenberg I, Katsarava Z, Sborowski A, Chernysh M, Osipova V, Tabeeva G, Yakhno N, Steiner TJ (2012). The prevalence of primary headache disorders in Russia: a countrywide survey. Cephalalgia.

[38] Yu S, Liu R, Zhao G, Yang X, Qiao X, Feng J, Fang Y, Cao X, He M, Steiner T (2012). The prevalence and burden of primary headaches in China: a population-based door-to-door survey. Headache.

[39] Stewart WF, Lipton RB, Celentano DD, Reed ML (1992). Prevalence of migraine headache in the United States. Relation to age, income, race, and other sociodemographic factors. JAMA.

[40] Stang PE, Osterhaus JT (1993). Impact of migraine in the United States: data from the National Health Interview Survey. Headache.

[41] Lipton RB, Stewart WF, Scher AI (2001). Epidemiology and economic impact of migraine. Curr Med Res Opin.

[42] Ertas M, Baykan B, Orhan EK, Zarifoglu M, Karli N, Saip S, Onal AE, Siva A (2012). One-year prevalence and the impact of migraine and tension-type headache in Turkey: a nationwide home-based study in adults. J Headache Pain.

[43] Parks SE, Housemann RA, Brownson RC (2003). Differential correlates of physical activity in urban and rural adults of various socioeconomic backgrounds in the United States. J Epidemiol Community Health.

[44] Drewnowski A, Specter SE (2004). Poverty and obesity: the role of energy density and energy costs. Am J Clin Nutr.

[45] Hsu CC, Lee CH, Wahlqvist ML, Huang HL, Chang HY, Chen L, Shih SF, Shin SJ, Tsai WC, Chen T, Huang CT, Cheng JS (2012). Poverty increases type 2 diabetes incidence and inequality of care despite universal health coverage. Diabetes Care.

[46] Linde M, Steiner TJ, Chisholm D (2015). Cost-effectiveness analysis of interventions for migraine in four low- and middle-income countries. J Headache Pain.

